# Right ventricular function following Surgical Aortic Valve Replacement (SAVR)

**DOI:** 10.1186/1532-429X-17-S1-P177

**Published:** 2015-02-03

**Authors:** Tarique A Musa, Akhlaque Uddin, Timothy A Fairbairn, Laura E Dobson, Ananth Kidambi, David P Ripley, Peter P Swoboda, Adam K McDiarmid, Bara Erhayiem, Pankaj Garg, Christopher D Steadman, Gerry P McCann, Sven Plein, John P Greenwood

**Affiliations:** Multidisciplinary Cardiovascular Research Centre & Leeds Institute for Cardiovascular and Metabolic Medicine, University of Leeds, Leeds, UK; Cardiovascular Sciences, National Institute of Health Research, University of Leicester, Cardiovascular Biomedical Research Unit, Leicester, UK

## Background

Right ventricular function is of prognostic importance in a variety of clinical settings but its complex anatomic geometry can pose a challenge to 2-dimensional imaging modalities. Right ventricular dysfunction is thought to occur following cardiac surgery and independently predicts adverse outcomes. However a clear mechanism for this dysfunction remains undefined.

We sought o accurately assess the effect of SAVR upon right ventricular function in patients treated for severe symptomatic aortic stenosis.

## Methods

All patients underwent an identical 1.5T CMR protocol (Intera, Phillips Healthcare, Best, The Netherlands or Avanto, Siemens Medical Systems, Erlangen, Germany). Multi-slice, multi-phase cine imaging was performed using a standard steady-state free procession pulse sequence in the short axis (8mm thickness, 0mm gap, 30 phases, typical field of view (FOV) 340mm) to cover the entire left and right ventricle. Late gadolinium enhancement (LGE) imaging (10-12 short axis slices, 10mm thickness, matrix 240×240, typical FOV 340mm) was performed following a Look-Locker inversion time scout, 10min after the administration of 0.2mmol/kg of gadoteric acid (Dotarem, Guerbet, Villepinte) or gadolinium-DTPA (Magnevist, Schering, Germany). Identical contrast agent was used at both study time-points.

## Results

53 SAVR patients (age 72.7±7.4years, 72% male, mean EuroSCORE II 1.52±0.95%) were studied before and 6 months after surgery. Six received a metallic prosthesis and the remaining 47 (89%) a tissue bioprosthesis. Fourteen (26%) received concomitant coronary bypass grafting, of which 6 involved use of the left internal mammary artery. For the group as a whole, the average bypass time was 105±48min and average cross clamp time 77±41min. The average length of stay in intensive care was 3.4±2.4 days. SAVR was associated with a significant decrease in RV ejection fraction and concomitant increase in indexed RVESV at 6 months, with no change in RV mass. However, in subgroup analysis of patients without LGE of the left ventricle at baseline, no significant change in RV function was seen following SAVR (p = 0.06).

## Conclusions

SAVR is associated with a significant reduction in right ventricular ejection fraction at 6 months mediated through an increase in end systolic volume. The presence of LGE may have the potential to identify patients at risk of post-operative RV dysfunction.

## Funding

This study was part-funded by the British Heart Foundation (BHF) (PG/11/126/29321).

GP McCann and CD Steadman have received support from the NIHR Leicester Cardiovascular BRU.

**Table 1:** RV changes following SAVR

RV	Baseline	6 months	p Value
EDVI (ml/m2)	78±17	78±16	0.90
ESVI (ml/m2)	33±10	37±10	< 0.01
EF (%)	58±8	53±9	< 0.01
Mass Index (g/m2)	16±4	15±4	0.15

